# Real-World Evidence for Risk Factors of Bruises and Fractures from Falls in Patients with Overactive Bladder: A Medical Record Analysis

**DOI:** 10.1155/2023/3701823

**Published:** 2023-12-27

**Authors:** Shigero Miyajima, Taisei Omaru, Tatsu Ishii, Hisatomi Arima, Yozo Shibata, Teruaki Izaki, Nobuhiro Haga

**Affiliations:** ^1^Department of Urology, Fukuoka University Chikushi Hospital, Chikushino, Fukuoka, Japan; ^2^Department of Preventive Medicine and Public Health, Faculty of Medicine, Fukuoka University, Fukuoka, Japan; ^3^Department of Orthopedic Surgery, Fukuoka University Chikushi Hospital, Chikushino, Fukuoka, Japan; ^4^Department of Urology, Fukuoka University, Fukuoka, Japan

## Abstract

**Aim:**

To identify the risk factors for bruises and fractures from falls in patients with overactive bladder (OAB).

**Methods:**

We evaluated 1136 patients with OAB and aged ≥50 years who visited our hospital. Age, sex, frequency of nocturnal urination, and urinary incontinence type were investigated in the 360 eligible patients. Patients were divided into three groups: those patients without falls (no-fall group), those with fall bruises (bruise group), and those with fall fractures (fracture group). The risk factors for bruises and fractures in patients with OAB were evaluated using the logistic regression analysis. In addition, association between the bruises or fractures from falls and the behavior around urination during the night was investigated.

**Results:**

The multivariate logistic regression analysis showed that female sex (odds ratio (OR) 2.888, *p* = 0.030) and nocturnal urination frequency ≥3 times/night (OR vs. ≤2 times/night, 2.940; *p* = 0.040) were significantly associated with bruises. Nocturnal urination frequency ≥3 times/night (OR vs. ≤2 times/night, 2.835; *p* = 0.026) and urge incontinence (OR 3.415, *p* = 0.016) were significantly associated with fractures. Behavior around urination during the night was significantly associated with fractures (*p* = 0.009).

**Conclusion:**

In the real-world clinical setting, increasing nocturnal urination frequency is a common risk factor for bruises and fractures. Also, female sex and urge incontinence were the risk factors for bruises and fractures, respectively. OAB patients with urge incontinence would especially require aggressive intervention to prevent fractures during night-time voiding.

## 1. Introduction

Overactive bladder (OAB) is a syndrome involving the urge to urinate with or without accompanying urinary incontinence. It is a common disease, and the patients typically have urinary frequency and nocturia [1–3]. The prevalence of OAB symptoms in adults aged ≥40 years was reported to be 12.4% in Japan [4] and 16.6% in France, Germany, Italy, Spain, Sweden, and the United Kingdom [5]. An influence of OAB on daily life was observed in more than half of the patients. OAB symptoms affect patients' quality of life (QOL) and mental health, and the urge to urinate had the most serious impact of all symptoms on the patients [6, 7].

More than 30% of people aged ≥65 years experience at least one fall per year [8, 9]. The proportion of OAB patients experiencing at least one fall per year ranges from 18.9% to 50.0%, overall suggesting an increased (1.3- to 2.3-fold) risk for falls [10].

Several groups, analyzing medical claims databases, have reported an association between OAB and falls and fractures [11–14]. However, their database analysis provided no clear evidence that bruises or fractures from falls were induced by OAB symptoms. Because the morbidity of OAB increases with age [15], frailty or sarcopenia might induce the falls. Therefore, real-world data concerning the association between OAB symptoms and falls inducing bruises or fractures is warranted. Findings of an association between them would increase the necessity of treatment for OAB.

The aim of our study was to identify the risk factors for bruises and fractures from falls in patients with OAB based on real-world data. In addition, understandably, OAB symptoms are strongly accompanied by the behavior around urination including going to and from a toilet. Thus, we also aimed to determine whether behavior around urination in OAB patients was associated with bruises and fractures.

## 2. Methods

This study was conducted in accordance with the tenets of the Declaration of Helsinki and was approved by the Fukuoka University Ethical Review Board (IRB No: C21-04-001). According to the current board rule, the acquisition of informed consent was exempted, since this study was retrospective. To collect the real-world data, we conducted a study of 1136 patients with OAB aged ≥50 years that had visited our hospital and were diagnosed by our urologists in the period from April 2005 to April 2019. We reviewed the medical records of all patients. The study was performed retrospectively with a medical record analysis. All patients who fell underwent X-rays and/or CT. According to the imaging findings, patients with fractures were categorized into the fracture group and patients without fractures were categorized into the bruise group. The types of urinary incontinence were classified using the International Continence Society (ICS) definition: urgency urinary incontinence, defined as the complaint of involuntary loss of urine associated with urgency; stress UI, defined as the complaint of involuntary loss of urine on effort or physical exertion or on sneezing or coughing; and mixed UI, defined as the complaint of involuntary loss of urine associated with urgency and with exertion, effort, sneezing, or coughing [16]. It also defines nocturia as waking to pass urine during the main sleep period [17]. To clarify the association between the OAB symptoms and falls, patients were divided into the following three groups: patients without falls (no-fall group), patients with bruises from falls (bruise group), and patients with fractures from falls (fracture group). The data on age, sex, frequency of nocturnal urination, and type of urinary incontinence were collected from the medical records.

In addition, to determine whether behavior around urination directly or indirectly led to bruises or fractures, the medical records were examined for the association of bruises or fractures with behavior including “going to the toilet” and “returning from the toilet.” Furthermore, the data were obtained to determine whether urination during the night led to bruises or fractures.

Inclusion criteria were defined by meeting of the following criteria: (1) patients with OAB aged ≥50 years and (2) patients evaluated for lower urinary tract symptoms with at least 3 months of observation. In the bruise and fracture groups, the inclusion criteria were patients with data of lower urinary tract symptoms or lower urinary tract function within 3 months before the occurrence of bruises or fractures.

Patients from whom we were unable to obtain sufficient data on age, sex, frequency of nocturnal urination, and urinary incontinence were also excluded. The patients' data from the first fall during the observation period were analyzed. Data from the second fall or later were excluded.

The primary endpoint of this study was to identify the risk factors for bruise or fractures from falls in patients with OAB. The secondary endpoint was to determine whether behavior around urination was associated with bruises or fractures.

Age, sex, frequency of nocturnal urination, and type of urinary incontinence were compared among the no-fall group, the bruise group, and the fracture group by the chi-squared test and analysis of variance (ANOVA). Behavior around urination which led to falls was compared between the bruise group and the fracture group by chi-squared test. Univariate and multivariate analyses were performed using a logistic regression model to determine the risk factors for bruises and fractures associated with falls in patients with OAB. All statistical analyses were conducted using the SPSS® (version 27) software (Chicago, IL, USA). *p* < 0.05 was considered to indicate a statistically significant difference.

## 3. Results

Of the 1136 patients with OAB, 136 patients had bruises and 109 patients had fractures from falls. No patients needed to undergo invasive treatments requiring a craniotomy or drainage procedure. We evaluated 360 patients, consisting of 291 patients in the no-fall group. After application of the exclusion criteria, there were 29 patients in the bruise group and 40 in the fracture group. With the current sample size (no fall group (*n* = 291) and fracture group (*n* = 40)), assuming that the average number of nocturnal urination was 2 (SD 1.5) in the no fall group, this study had more than 80% power to detect a 0.71 or more increase in the average number of nocturnal urination (i.e. ≥2.71) in the fracture group. Among those patients, three patients visited our clinic after a second fall. Two patients had only bruises, and one patient had only fractures in both the first and second falls. We analyzed the data from the first fall only for these three patients and excluded the data from the second fall.

Patient characteristics and symptoms are listed in Table 1. There were 234 males and 57 females aged 77.3 ± 8.4 in the no-fall group, 18 males and 11 females aged 77.2 ± 9.1 in the bruise group, and 28 males and 12 females aged 78.9 ± 7.2 in the fracture group (Table 1). The frequency of nocturnal urination was 2.3 ± 1.3, 2.8 ± 1.1, and 3.2 ± 1.5 in the no-fall group, bruise group, and fracture group, respectively. Twenty-two, 5, and 9 patients had urge incontinence, 25, 2, and 2 patients had stress urinary incontinence, and 1, 3, and 1 patients had mixed urinary incontinence in the no-fall group, bruise group, and fracture group, respectively (Table 1). Significant differences in sex, nocturnal urination frequency, and the number of patients with urge incontinence and mixed urinary incontinence were observed among the three groups. No significant difference in age or the percentage of patients with stress urinary incontinence was detected (Table 1).

**Figure 1:**
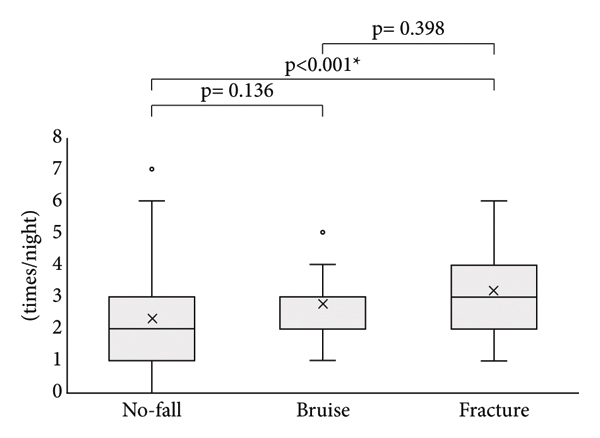
Comparison of nocturnal urination frequency among no-fall, bruise, and fracture groups. Nocturnal urination frequency was compared among no-fall, bruise, and fracture groups. ^*∗*^Statistically significant *p* < 0.05.

**Figure 2:**
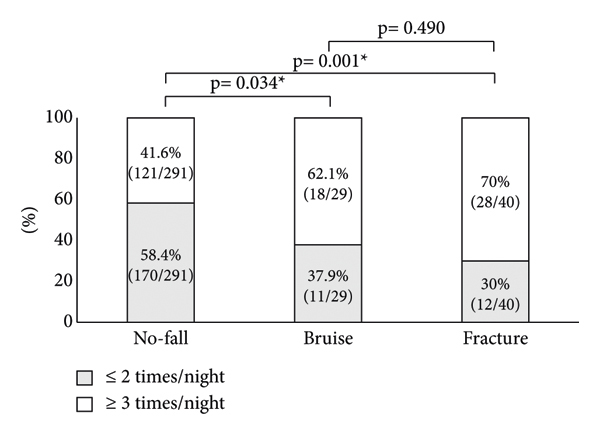
Comparison of nocturnal urination frequency (≤2 times vs. ≥3 times) in patients among no-fall, bruise, and fracture groups. Gray boxes indicate patients who urinate ≤2 times/night and white boxes indicate patients who urinate ≥3 times/night. ^*∗*^Statistically significant *p* < 0.05.

**Figure 3:**
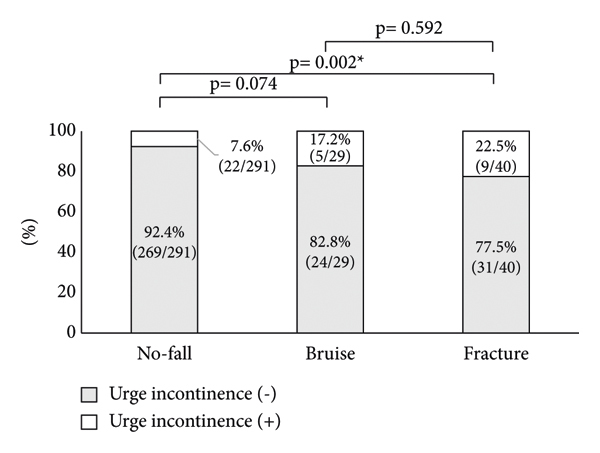
Comparison of the percentage of patients with urge incontinence among no-fall, bruise, and fracture groups. Gray boxes indicate patients without urge incontinence and white boxes indicate patients with urge incontinence. ^*∗*^Statistically significant *p* < 0.05.

Next, the association between the OAB symptoms and falls was investigated. Significant differences in nocturnal urination frequency were observed between the no-fall and fracture groups, but no significant difference was observed between the no-fall and bruise groups and between bruise and fracture groups (Figure 1). The percentage of patients who urinate 2 times or less during the night or 3 times or more differed in each group. Significant differences in nocturnal urination frequency (≤2 times/night vs. ≥3 times/night) were observed between the no-fall and bruise groups and between no-fall and fracture groups, but no significant difference was observed between bruise and fracture groups (Figure 2). A significant difference in the percentage of patients with urge incontinence was observed between the no-fall group and fracture group, but no significant difference was detected between the no-fall group and bruise group and between the bruise group and fracture group (Figure 3).

Furthermore, risk factors for bruises and fractures from falls associated with OAB symptoms were investigated. Female (OR vs. male, 2.888; *p* = 0.030) and nocturnal urination frequency ≥3 times/night (OR vs. ≤2 times/night, 2.940; *p* = 0.040) were significantly associated with bruises in falls (Table 2). Nocturnal urination frequency ≥3 times/night (OR vs. ≤2 times/night, 2.835; *p* = 0.026) and urge incontinence (OR, 3.415; *p* = 0.016) were significantly associated with fractures from falls (Table 3).

**Figure 4:**
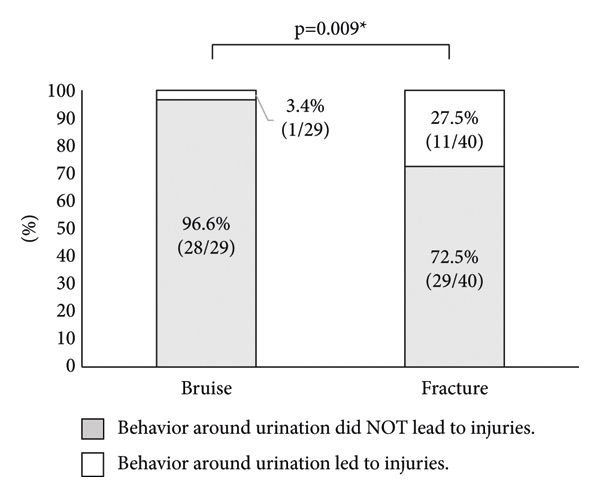
Comparison of whether behavior around urination during the night led to injuries in patients among bruise and fracture groups. Gray boxes indicate patients whose behavior around urination did not lead to injuries during the night. White boxes indicate patients whose behavior around urination led to injuries during the night. ^*∗*^Statistically significant *p* < 0.05.

In addition, behavior around urination that directly or indirectly led to bruises or fractures was investigated. Behavior around urination led to bruises from falls in 13.7% of patients (4/29) and fractures in falls in 30% of patients (12/40). No significant difference was observed between these two groups (*p* = 0.115). On the other hand, behavior around urination during the night led to bruises from falls in 3.4% of patients (1/29) and fractures from falls in 27.5% of patients (11/40). Behavior around urination during the night was significantly associated with fractures (*p* = 0.009) (Figure 4).

**Figure 5:**
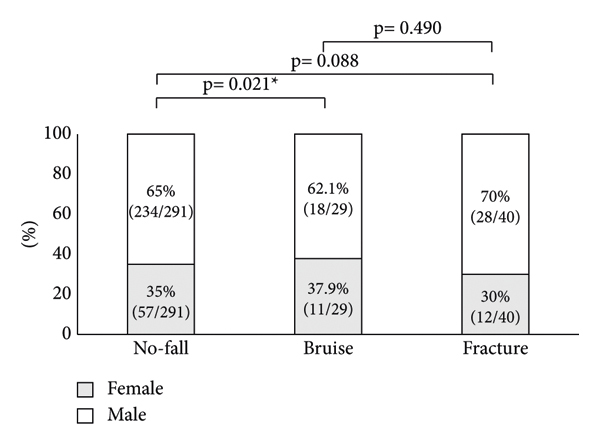
Comparison of the percentage of patients with gender differences among no-fall, bruise, and fracture groups. Gray boxes indicate female patients and white boxes indicate male patients. ^*∗*^Statistically significant *p* < 0.05.

At last, in the subgroup analysis by gender, a significant difference in the percentage of patients with gender differences was observed between the no-fall group and bruise group, but no significant difference was detected between the no-fall group and fracture group and between the bruise group and fracture group (Figure 5).

## 4. Discussion

In this study, we investigated the risk factors for bruises and fractures from falls in patients with OAB based on real-world data through the review of the medical records. Previous studies examined the association of OAB symptoms and falls or fractures separately based on the data from large databases [11–14]. Injuries from falls range from the slight bruising to serious fractures. We examined each risk factor for bruises and fractures from falls in OAB patients in this cohort. Surprisingly, this study revealed that there were some differences between the risk factors for bruises and fractures. Female sex was a unique risk factor for bruises, and urge incontinence was a unique risk factor for fractures. The increase in nocturnal urination frequency was a common factor.

As mentioned above, our results revealed that increased nocturnal urination frequency was a common risk factor for bruises and fractures. In addition, OAB patients who urinate ≥3 times during the night had approximately 2.9-fold and 2.8-fold higher risks for bruises and fractures, respectively, compared with patients who urinate ≤2 times during the night. Increased nocturnal urination frequency was associated with an increased risk for fractures and falls, which is similar to the findings of other meta-analysis or previous studies using medical databases [18–20]. As one of the causes of the increase of bruises and fractures due to the increase in nocturnal urinary frequency, a decrease in concentration due to a sleep disorder might be involved [21, 22].

We suggest that female sex is a risk factor for bruises from falls. A systematic review reported that the risk for falls is higher in females than males [10]. Muscle weakness in the lower body is a risk factor for falls [23]. Elderly females have a high risk for falls because the lower extremity muscles are weaker in elderly females than males and balance disability is often observed in elderly females [24]. Muscle weakness and abnormal balance in patients were not evaluated in this study, but they are presumed to be associated with more falls in females. Thus, although the morbidity of OAB was greater in females [5, 10], OAB symptoms themselves might not be involved in the increase of bruises in the present study.

This study demonstrated that urge incontinence resulted in an approximately 3.4-fold increase in the risk for fractures from falls. Other studies also reported that urge incontinence is a risk factor for fractures [25–27]. In addition, urge incontinence significantly decreases QOL in patients with OAB [7]. Thus, OAB patients with urge incontinence might require aggressive intervention from the viewpoint of preventing fractures in falls. Moreover, we showed that fractures from falls during the behavior around urination during the night have a significantly higher incidence than bruises. Therefore, it would be important for OAB patients to have an improved environment such as installing handrails around a toilet.

There are some limitations in this study. First, for the analysis of patients with bruises, we analyzed only patients who visited our hospital. Thus, the number of patients with bruises might be underestimated, since we did not include the data from patients who did not visit our hospital. Second, there were no data regarding BMI, bone density, muscle weakness, balance, visual acuity, or cognitive deficiency in patients, since this was a retrospective study. Last, we did not evaluate OAB symptoms in patients using some indices such as the overactive bladder symptom score (OABSS) [28]. However, it might not affect the present results because the physicians participating in this study thoroughly evaluated the patients' OAB status.

## 5. Conclusions

In this study, female sex and urge incontinence were found to be risk factors for bruises and fractures from falls, respectively, in OAB patients. On the other hand, a common risk factor for bruises and fractures was an increase in nocturnal urination frequency. In addition, behavior around urination during the night is associated with fractures from falls. Thus, female OAB patients with nocturia and urge incontinence would require aggressive intervention. It is also necessary for such patients to pay attention to the behavior around urination during the night to prevent the fractures in the falls.

## Figures and Tables

**Table 1:** Clinical characteristics of no-fall, bruise, and fracture groups.

	No-fall	Bruise	Fracture	*p* value
Number of patients	291	29	40	
Age (mean ± SD) (y.o.)	77.3 ± 8.4	77.2 ± 9.1	78.9 ± 7.2	0.536
Sex (male/Female)	234/57	18/11	28/12	0.035
Nocturnal urination frequency	2.3 ± 1.3	2.8 ± 1.1	3.2 ± 1.5	<0.001
Type of UI				<0.001
Urge UI (%)	22 (7.6)	5 (17.2)	9 (22.5)	0.005
Stress UI (%)	25 (8.6)	2 (6.9)	2 (5)	0.716
Mixed UI (%)	1 (0.3)	3 (10.4)	1 (2.5)	<0.001

SD, standard deviation; UI, urinary incontinence.

**Table 2:** Risk factors for bruises in falls in overactive bladder patients.

	Univariate analysis			Multivariate analysis		
	OR	95% CI	*p* value	OR	95% CI	*p* value
Age	0.997	0.952–1.043	0.882	0.996	0.946–1.049	0.883
Sex (females vs males)	2.320	1.047–5.142	0.038	2.888	1.110–7.515	0.030
Nocturnal urinary frequency (≤2 times vs. ≥3 times)	1.999	0.916–4.363	0.082	2.940	1.052–8.214	0.040
Urge incontinence	2.090	0.743–5.878	0.162	2.058	0.619–6.850	0.239

OR, odds ratio; CI, confidence interval.

**Table 3:** Risk factors for fractures in falls in overactive bladder patients.

	Univariate analysis			Multivariate analysis		
	OR	95% CI	*p* value	OR	95% CI	*p* value
Age	1.024	0.982–1.068	0.264	1.020	0.968–1.075	0.452
Sex (females vs males)	1.759	0.843–3.671	0.132	2.293	0.924–5.687	0.073
Nocturnal urinary frequency (≤2 times vs. ≥3 times)	3.278	1.603–6.703	0.001	2.835	1.136–7.073	0.026
Urge incontinence	3.550	1.502–8.389	0.004	3.415	1.257–9.279	0.016

OR, odds ratio; CI, confidence interval.

## Data Availability

The data that support the findings of this study are available from the corresponding author upon reasonable request.

## Additional Points

*What's Known?* Some studies have reported an association between OAB and falls and fractures. They just analyzed the responses to questionnaires or medical claims databases. There have been no studies examining whether behavior around urination including “going to the toilet” and “returning from the toilet.” are associated with falls and fractures in overactive bladder patients. *What's New?* In this study, female sex and urge incontinence were found to be risk factors for bruises and fractures from falls, respectively, in OAB patients based on real-world data. On the other hand, a common risk factor for bruises and fractures was an increase in nocturnal urination frequency. In addition, behavior around urination during the night is associated with fractures from falls.
